# Lithium Antiperovskite-Derived Glass Solid Electrolytes

**DOI:** 10.1021/acsmaterialslett.4c02578

**Published:** 2025-02-25

**Authors:** Emily Milan, Gregory J. Rees, Aaron Phillips, Cristian Cano, Yi Wei, Hua Guo, Steve Feller, Mauro Pasta

**Affiliations:** † Department of Materials, University of Oxford, Oxford OX1 3PH, U.K.; ‡ Department of Physics, Coe College, Cedar Rapids, Iowa 52402, United States

## Abstract

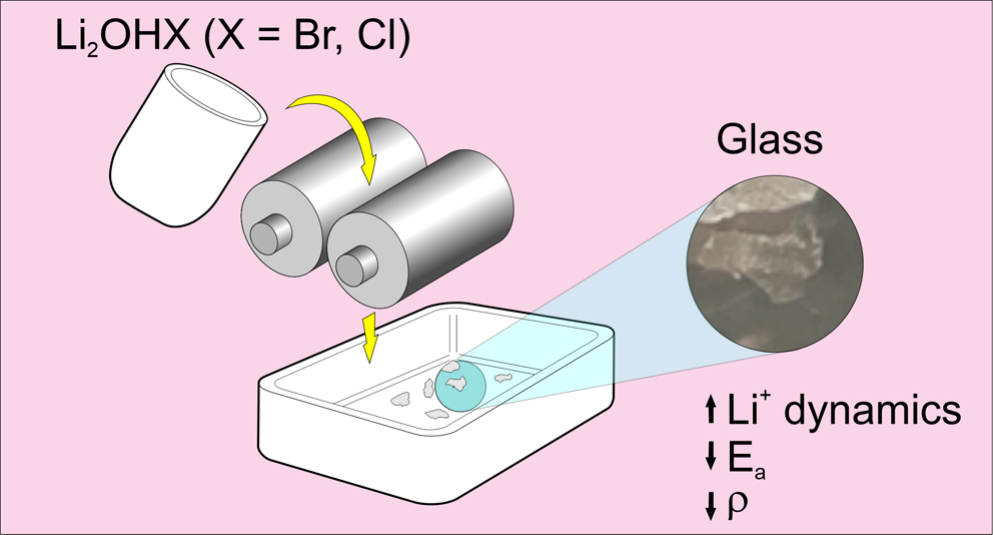

In this paper, we report the synthesis of Li_2_OHX (X = Br, Cl)-based glasses. These glasses were found to be challenging to synthesize, requiring extreme cooling rates achievable only by a twin-roll quench process. As has been speculated for antiperovskite-derived glasses, indications of improved lithium-ion dynamics are observed. Notably, spin–lattice relaxation nuclear magnetic resonance spectroscopy reveals a higher hopping frequency and significantly lower activation energy for Li_2_OHBr glasses (0.29 eV) compared to the crystalline Li_2_OHBr (0.39 eV). This may be attributable to the increased free volume in the glass samples (ρ_glass_/ρ_cryst_ = 0.83) and a reduced ionic interaction of lithium ions with the glass structure. Despite these promising findings, the glasses were found to be unstable under pressure and crystallized in attempts to produce bulk samples for impedance measurements.

In the drive to reach net-zero, energy storage is a crucial area of research. Solid-state batteries have been proposed as a solution to meeting increasing demands, thanks to the potential increases in energy density which could be obtained through their use with a lithium metal anode.^1−3^ Among the solid-electrolytes being investigated for this application, are the Li_2_OHX (X = Cl, Br) antiperovskites.^4−10^ These host a wealth of advantages including a wide electrochemical stability window, stability with lithium metal, low electronic conductivity, relatively low cost and nontoxicity.^11−13^ However, they are limited by their suboptimal ionic conductivity (∼10^–6^ S cm^–1^), which has often been attributed to high grain boundary resistances.^7,9,14,15^ A potential approach to retaining the desirable properties of the antiperovskite electrolytes while simultaneously attempting to increase the ionic conductivity, is to make glassy samples. Although the exact definition of a glass can be debated, a sufficient description for most instances is a homogeneous, isotropic and noncrystalline material, free from any internal phase boundaries.^16^ Instead of conduction mechanisms relying on intrinsic or extrinsically doped mobile defects, glasses typically incorporate a large amount of free volume in their structure, which is available as sites for migrating ions to hop into, and may result in improved ionic conductivity. Additionally, the homogeneous nature of glasses mean that ionic conduction is isotropic over a long length scale, and avoids the detrimental impacts of grain boundaries. Other potential advantages proposed for glass electrolytes include improved resistance to lithium filament growth, better capacity retention, and enhanced rate performance compared to their crystalline counterparts.^17,18^ Glass electrolytes have been a topic of interest since the 1980s, with extensive research into lithium thiophosphate (LPS) glasses^19−26^ and lithium phosphorus oxynitride (LiPON) glasses.^27−33^ Recently, interest into novel glass electrolytes has continued, with exciting reports of amorphous electrolytes exhibiting impressive superionic conduction.^34−37^

In principle, any material system can be vitrified by quenching from the molten state to below its glass transition temperature, *T*_g_, while suppressing nucleation of crystals.^38^ However, unlike LiPON and LPS which derive from more conventional glasses consisting of “network formers” (PO_4_^3–^ and PS_4_^3–^ respectively) and lithium modifiers, the antiperovskites do not contain any natural network formers and are not conducive to satisfying the random network theory rules proposed by Zachariasen back in 1932.^39^ Additionally, despite the energy and cathode coprocessing advantages offered by the low melting points of these antiperovskites (≤300 °C), the low temperatures can cause challenges in producing the high cooling rates necessary for quenching as is traditionally done to kinetically lock the glass phase in. As such, producing antiperovskite-derived glasses is expected to be challenging.

Between 2014 and 2017, and more recently in 2024, Braga et al. published a series of papers claiming to have made glassy “antiperovskite” electrolytes based on a lightly doped Li_3_OCl composition, using an unconventional wet synthesis approach.^40−44^ An ionic conductivity of 25 mS cm^–1^ at 25 °C was reported for Li_2.99_Ba_0.005_OCl, the highest of any solid electrolyte at the time. In the reported synthesis, water is added to LiCl, Ba(OH)_2_ and LiOH precursors to create a paste, which is then dried at high temperatures. This is followed by manipulation in air which is claimed to be necessary to promote formation of the glassy phase. The work received notable attention due to its impressive claims. However, criticism soon followed, and the work is now regarded as controversial.^45,46^ Notably, Hanghofer et al. conducted a study to investigate the reported synthesis procedure.^45^ In their work, degradation of the supposed “Li_3_OCl” to Li_2_CO_3_ and LiCl·*x*H_2_O was found. Hydrated lithium chloride is amorphous, matches the differential scanning calorimetry (DSC) results observed by Braga et al., and exhibits a high ionic conductivity, explaining the results found by Braga and rendering the glass reports unreliable.

Nevertheless, the claims made by Braga et al. inspired several computational studies into related antiperovskite-derived glasses.^47−49^ Heenen et al. created Li_3_OCl glass models through simulated quenches from 1200 K to 300 K and calculated resulting ionic conductivities using a molecular dynamics approach.^47^ Ionic conductivities of 1–10 mS cm^–1^ were found for the glassy Li_3_OCl, much higher than that of the crystalline form, and an activation energy of 0.42 eV. This improvement in ionic conductivity was attributed to a predicted 13% volume increase in the glassy state, and a shift from vacancy conduction to Li-ion migration. Another computational study looked at sodium analogues, predicting glassy Na_3_OCl to have an excellent ionic conductivity of ∼16 mS cm^–1^ at 300 K. This was improved further by the creation of glassy Na_2_OHCl, thanks to the introduction of an −OH paddlewheel mechanism.^49^ Smith and Siegel have also proposed that advantageous paddlewheel mechanisms could be active at low temperatures in glasses with limited network-forming ability,^50^ a prediction that is expected to extend to Li_2_OHX (X = Cl, Br) antipervoskites as well. These computational findings suggest that, despite the controversial reports surrounding experimental lithium oxyhalide glasses, the lithium-ion dynamics in an Li_2_OHX (X = Cl, Br) glass may be desirable and should be investigated.

In this paper, the synthesis of glassy lithium hydroxyhalide antiperovskites are investigated using a melt-quench approach. This study aims to resolve the controversies arising from earlier reports of antiperovskite glasses by synthesizing real antiperovskite glasses, and assessing whether they exhibit improved lithium-ion dynamics compared to their crystalline counterparts. Diffraction and thermoanalytical techniques are employed to confirm the glassy state of successful samples. Subsequent chemical and structural characterization is utilized to understand more about these novel glasses. Lithium-ion dynamics in Li_2_OHBr glasses are probed through spin–lattice relaxation (SLR) nuclear magnetic resonance (NMR) spectroscopy measurements, to establish whether any improvement is observed over the crystalline antiperovskite phase. In spite of promising findings, the inherent instability of these glasses against crystallization may limit their practical application in solid-state batteries, and future work should therefore focus on strategies to enhance their stability.

Synthesis was investigated for the bromide (Li_2_OHBr) and chloride (Li_2_OHCl) antiperovskite compositions. Additionally, a mixed halide composition with the stoichiometry Li_2_OHBr_0.5_Cl_0.5_ was investigated, with the anticipation that the increased disorder resulting from the mixed-anion site may aid in glass-forming ability.

Stoichiometric anhydrous LiCl, LiBr and LiOH were ground together using a mortar and pestle, and heated to the liquid state at 350 °C in an alumina crucible for 45 min under an argon atmosphere. The crucibles were removed from the furnace to cool to room temperature, and the resulting sample ground into a powder using a mortar and pestle. This material constitutes the crystalline antiperovskites referenced throughout the paper. For glass synthesis, the samples were remelted to undergo the quenching process. Formation of the glassy state was found to be very challenging, and sufficient cooling rates could only be achieved through use of a twin-roll quench process, conducted inside of a nitrogen-filled glovebox^51^ (see Supporting Information Note 1). In this approach, the molten sample is poured over the face of two spinning steel rollers, each 10 cm in diameter. Powerful motors force the material through the gap between the rollers, into a collection bin beneath. The rapid increase in sample surface area which results, enables much higher cooling rates (∼5 × 10^5^ K/s) to be attained than by a conventional quenching process.

Flakes of material with varying crystallinity were produced from this process. Polycrystalline samples are expected to be opaque due to the scattering of light by defects such as grain boundaries, and so optically transparent flakes were separated as promising samples. Photographs showing examples of these transparent and opaque flakes can be seen in Figures 1a and b.

**Figure 1:**
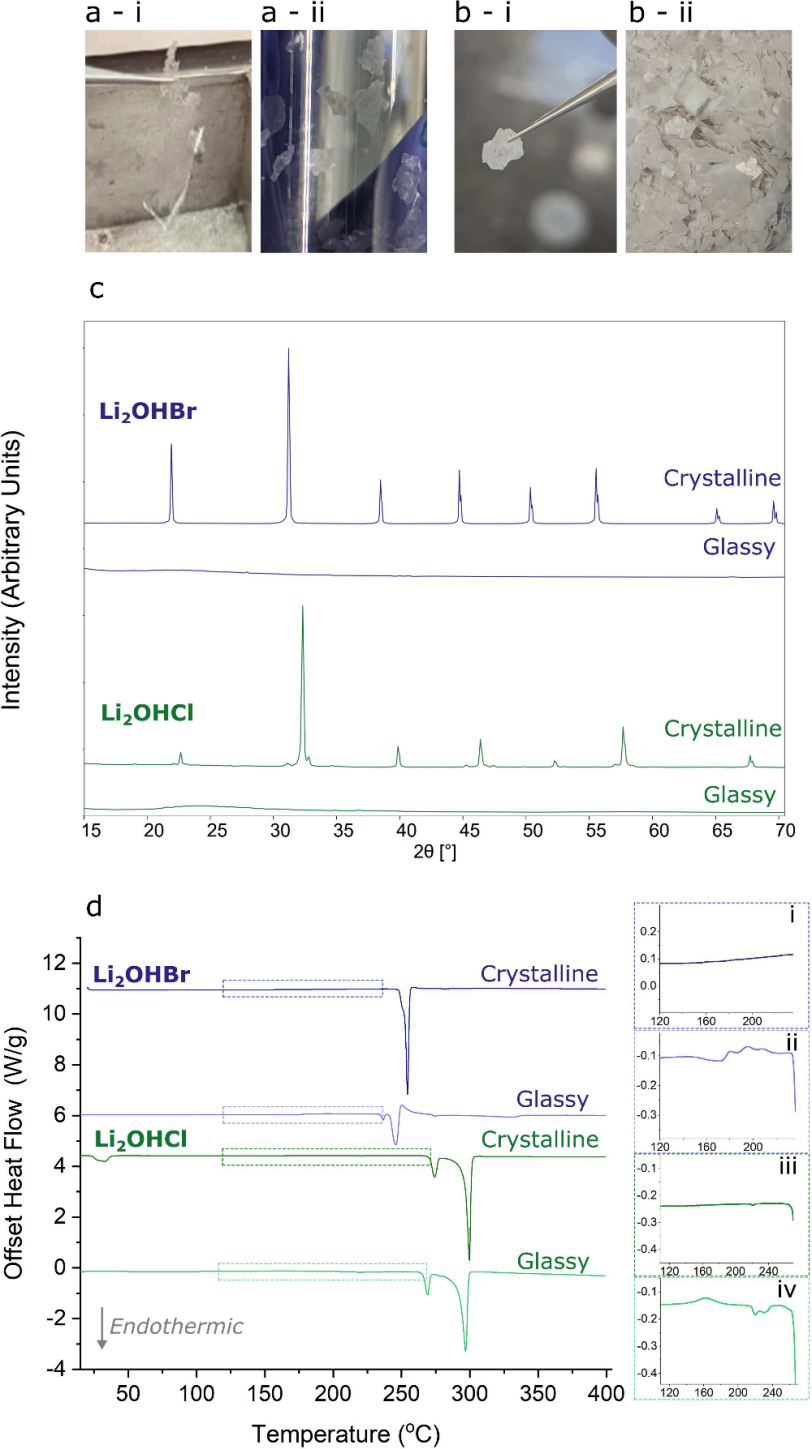
Proof of glass synthesis. a) Photographs of transparent “glassy” Li_2_OHBr flakes (i) held by tweezers and (ii) stored in a vial. b) Similar photographs, this time showing opaque examples of flakes. c) XRD patterns of Li_2_OHBr and Li_2_OHCl glasses showing the absence of the characteristic Bragg peaks seen in the crystalline homologues. d) DSC measurements of crystalline and glassy Li_2_OHBr and Li_2_OHCl on the first heating cycle. The insets (i–iv) show glass transition events are present only in the glassy samples.

From optimization of the twin-roll quench conditions, fully transparent flakes of Li_2_OHBr and Li_2_OHCl could be obtained, with greater success from the bromide compositions. Glasses should not contain long-range order, and so X-ray diffraction (XRD) measurements were taken on crushed samples to ascertain whether this was the case. The crystalline samples exhibit Bragg peaks corresponding to the cubic antiperovskite phase, with additional peaks observed in the crystalline Li_2_OHCl corresponding to the orthorhombic antiperovskite, as is expected for the chloride composition below ∼35 °C.^5,52,53^ Supporting Pawley refinements are included in Figure S1 (Table S1). As shown in Figure 1c, no Bragg peaks are observed in the completely transparent Li_2_OHBr and Li_2_OHCl flakes. These findings support that the transparent samples do not have a crystalline structure.

Another important feature of melt-quench glasses is that they exhibit an endothermic second-order transition known as the glass transition temperature, *T*_g_. This manifests as discontinuities in derivative thermodynamic properties such as heat capacity and thermal expansion.^16^ The glass transition arises as a result of the rapid decrease in viscosity and corresponding increase in mobility of the atoms in a structure as the temperature is increased above its *T*_g_ (ν ≈ 10^12^ Pa.s^16^). A crystallization temperature, *T*_c_, is typically found above this temperature, at which point the material undergoes an exothermic transition from the disordered state to the thermodynamically preferable crystalline state. It is worth noting though, that spontaneous crystallization is possible below these temperatures, particularly in unstable glasses such as these.

DSC was conducted on transparent Li_2_OHBr and Li_2_OHCl samples to observe whether glass transitions occur upon heating, shown in Figure 1d. In both instances, features indicative of a glass transition can be seen in the samples’ responses, confirming the presence of a glass phase. These features are absent in measurements taken on crystalline samples.

In Li_2_OHCl, a small endothermic peak corresponding to the orthorhombic-to-cubic phase transition can be seen in the crystalline sample at ∼30 °C.^5,52,53^ This is absent in the glass samples since they do not contain any orthorhombic Li_2_OHCl. In the glass, a small exothermic hump occurs just above 150 °C, visible in the zoomed-in region shown in inset iii of Figure 1d. This corresponds to sub-*T*_g_ relaxation, whereby some structural rearrangements to a more stable environment occur. Around 225 °C, a small endothermic slope signifies the onset of the glass transition. This is immediately followed by exothermic responses corresponding to subsequent rearrangement and crystallization occurring. Multiple exothermic events appear to occur, which may be indicative of multiple glass phases existing, or could be a result of a series of crystallization steps occurring from a single glass phase. Above this temperature, the material behaves as the crystalline Li_2_OHCl, albeit with a small shift to lower temperatures (≈5 °C).

As in the chloride glass, features indicative of a glass transition are seen in measurements of the bromide glass samples from ∼150 °C. Similarly, various exothermic crystallization responses appear to be occurring above the glass transition. The absence of a “working range” between the glass transition and crystallization temperatures eliminates any possibility of processing these glasses in the viscous state, as has been previously demonstrated for other glass electrolytes.^34,54,55^ For the bromide glass, the behavior of the profile after crystallization is noticeably different from that of the pristine crystalline material: the endothermic peak observed at 254 °C in crystalline measurements is replaced with endothermic peaks at 237 °C and 246 °C. It was not possible to capture what was happening through ex-situ experiments, due to the narrow temperature ranges of interest. Nevertheless, the samples are no longer glassy on subsequent heating and cooling cycles, apparent from the disappearance of the glass transition (Figure S2).

Although it was possible to obtain pure Li_2_OHBr and Li_2_OHCl glass samples, some crystalline material was typically present, discussed further in Supporting Information Note 2, even after optimizing the synthesis conditions. Synthesis was most successful using the Li_2_OHBr composition, and so subsequent measurements focused on Li_2_OHBr glass, selected based on appearance.

Scanning electron microscopy (SEM) images of the surface and cross-section (prepared using a plasma focused-ion beam) of Li_2_OHBr glass flakes were taken, however no phase contrast was observed (Figure 2a and b). Corresponding energy-dispersive X-ray (EDX) spectroscopy mapping was conducted to see whether any chemical segregation could be identified. For example, a mixture of two glass phases, or the possibility to distinguish between crystalline and glassy regions. The elemental concentrations were found to be homogeneous, indicating that no significant variations in stoichiometry occur (Figure S4).

**Figure 2:**
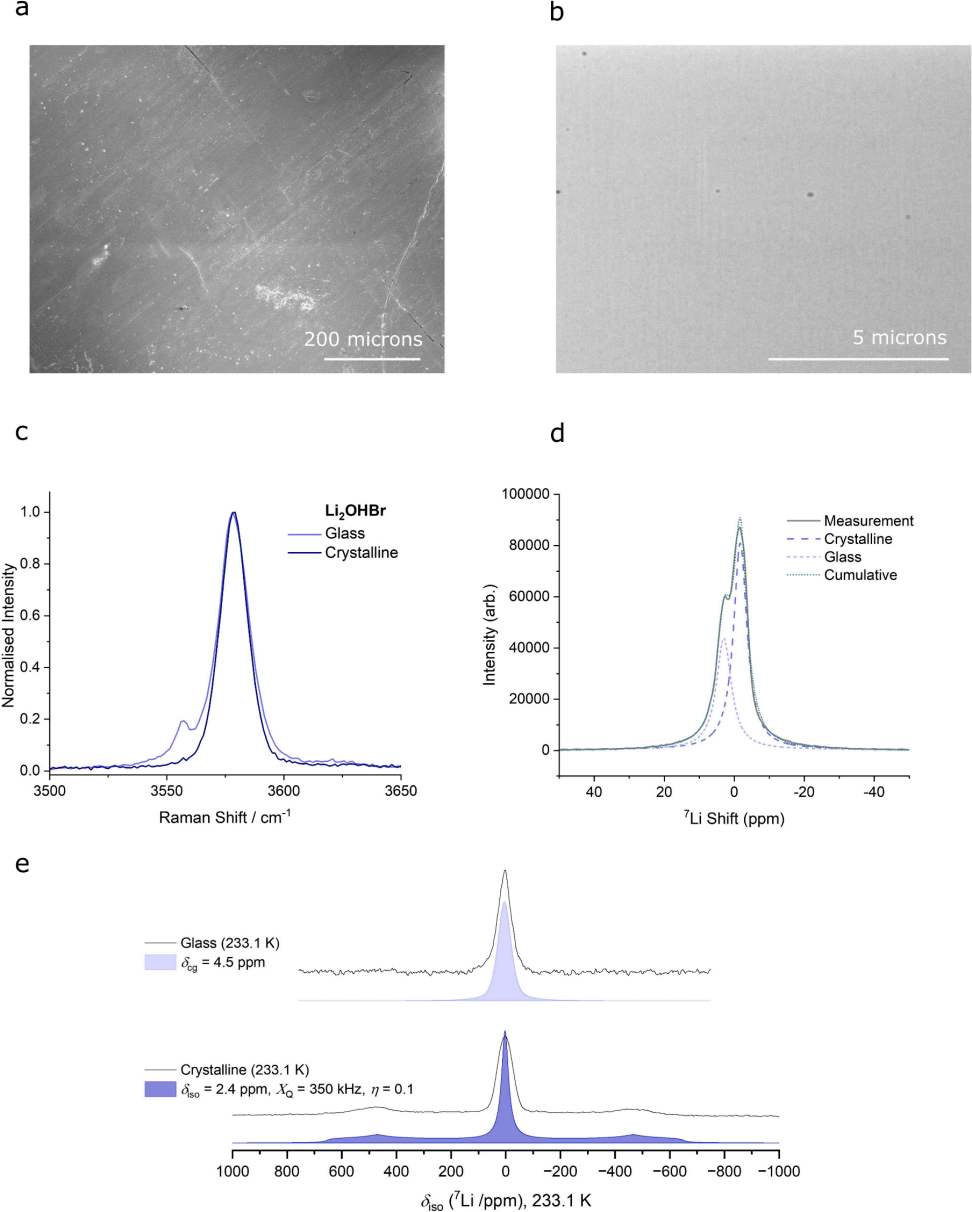
Structural and chemical characterization. a) Surface SEM imaged of a Li_2_OHBr glass flake. The rolling direction of the twin-roll quench process can be seen to go from bottom left to top right. Small cracks can be seen in various locations. Small amounts of surface contamination appear as bright specs. b) SEM image of a cross-section of a Li_2_OHBr glass flake prepared using a plasma focused-ion beam. Small isolated pores can be seen in various locations. c) Raman spectroscopy of glassy and crystalline Li_2_OHBr measured between 3500 and 3650 cm^–1^. d) ^7^Li MAS NMR line shape obtained from glass-ceramic Li_2_OHBr at 393 K, showing a glass peak centered on 2.9 ppm and a crystalline peak at −1.6 ppm. e) Static ^7^Li NMR of glass and crystalline Li_2_OHBr. Satellites are only observed in the crystalline material due to its high symmetry local structure.

Glasses typically incorporate free space into their structure, and so the antiperovskite glasses are expected to have a lower density than their crystalline counterparts. The density of pure Li_2_OHBr glass flakes was measured using a sink-float method. Very little porosity can be seen in PFIB cross sections of transparent glass flakes, as were selected for these measurements (Figure S5). As such, the porosity can be assumed to be negligible. The measurements indicate a density of 2.31 g/cm^3^ ± 0.05 g/cm^3^ for the Li_2_OHBr glass. This is 83% of the theoretical density of crystalline Li_2_OHBr (2.78 g/cm^3^). This impressive observed decrease in density may be beneficial to the lithium dynamics in the material, since the increased free volume can act as potential sites for lithium ions to hop into during migration. Relationships between decreased glass density and increased ionic conductivity have been reported previously,^56−58^ and in Heenen’s computational study of Li_3_OCl glasses, enhanced ion mobilities were observed in ensembles quenched to lower densities.^47^

Raman spectroscopy is a technique probing short- and medium-range order, often employed in the study of glasses.^59−61^ However, only the O–H bond stretching vibration is active in the antiperovskite compositions studied here, making it of limited use. The O–H environment in the Li_2_OHBr glass appears very similar to the Li_2_OHBr antiperovskite, showing a dominant peak at 3579 cm^–1^ (Figure 2c). A small peak is also present at 3555 cm^–1^, indicating the presence of another environment in the glassy state. Additionally, a slight increase in broadening can be seen in the glassy state, maybe suggesting a broader range of environments. The other glass compositions, Li_2_OHCl and Li_2_OHCl_0.5_Br_0.5_, did not exhibit any additional peaks, and broadening was not apparent (Figure S6).

^7^Li magic angle spinning (MAS) NMR spectroscopy measurements of Li_2_OHBr mixed glass-ceramic samples were taken at a range of temperatures between 303 and 393 K. Peaks corresponding to the crystalline and glassy phases can be deconvoluted, especially apparent at higher temperatures due to the motional narrowing that occurs (Figure 2d, Figure S7). The peak corresponding to the crystalline phase, assigned using a pure crystalline sample, occurs at a more negative shift than the glass peak. The more positive shift in the glass phase could be an indication of longer bond lengths. The increased shielding, and reduced ionic interaction implied from this, may be beneficial to lithium mobility in the glassy state.

Low temperature (233.1 K) static ^7^Li NMR of glass and crystalline samples were taken (Figure 2e). In the crystalline material, the high symmetry local structure creates distinct electric field gradients (EFGs), leading to quadrupolar interactions with the nuclear quadrupole moment, and hence the appearance of satellite peaks. In the glass, the distribution of chemical shifts and quadrupole coupling constants causes broadening, which merges the satellite peaks into the baseline. This further supports that the samples measured correspond to a glassy, disordered state.

The potential for improved ionic conductivity was the original motivation for synthesizing the antiperovskite glasses. To obtain an indication of the lithium dynamics in the Li_2_OHBr glass, SLR-NMR measurements were taken as a function of temperature.

The relaxation rate, R_1ρ_, is sensitive to Li-ion jump rates, which are proportional to the applied locking field in the rotating frame (kHz, 10^5^ s^–1^, milliTesla). Consequently, relaxation rates are influenced by fluctuations in the local nuclear environments that occur on time scales inversely proportional to the applied field (10 kHz). The temperature-dependent R_1ρ_ values pass through a maximum when the fluctuation time scales commute with the applied field. The resultant Lorentzian peaks are typically asymmetrical due to correlation effects, such as disorder, affecting the low-temperature flank. The R_1ρ_*T* curves can be fitted to the Bloembergen, Purcell, and Pound (BPP) model (, where β accounts for the asymmetry).^62^ In the high-temperature regime of a three-dimensional isotropic ion conductor, where ω_0_τ ≪ 1, the activation energy (*E*_a(HT)_) for long-range ion motion does not suffer from these adverse effects and so is typically quoted.^63^

The R_1ρ_ rate peaks for glass and crystalline Li_2_OHBr are plotted in Figure 3. In both instances, two peaks (R_1ρ(max)_) of very similar activation energy can be seen, as was observed for crystalline Li_2_OHCl by Wilkening et al.^64^ These may be attributed to bulk Li-ion diffusion through the antiperovskite, and the onset of the hydroxyl-ion paddlewheel rotations, which has been the topic of extensive investigation, and is believed to help facilitate lithium ion motion.^53,64−67^

**Figure 3:**
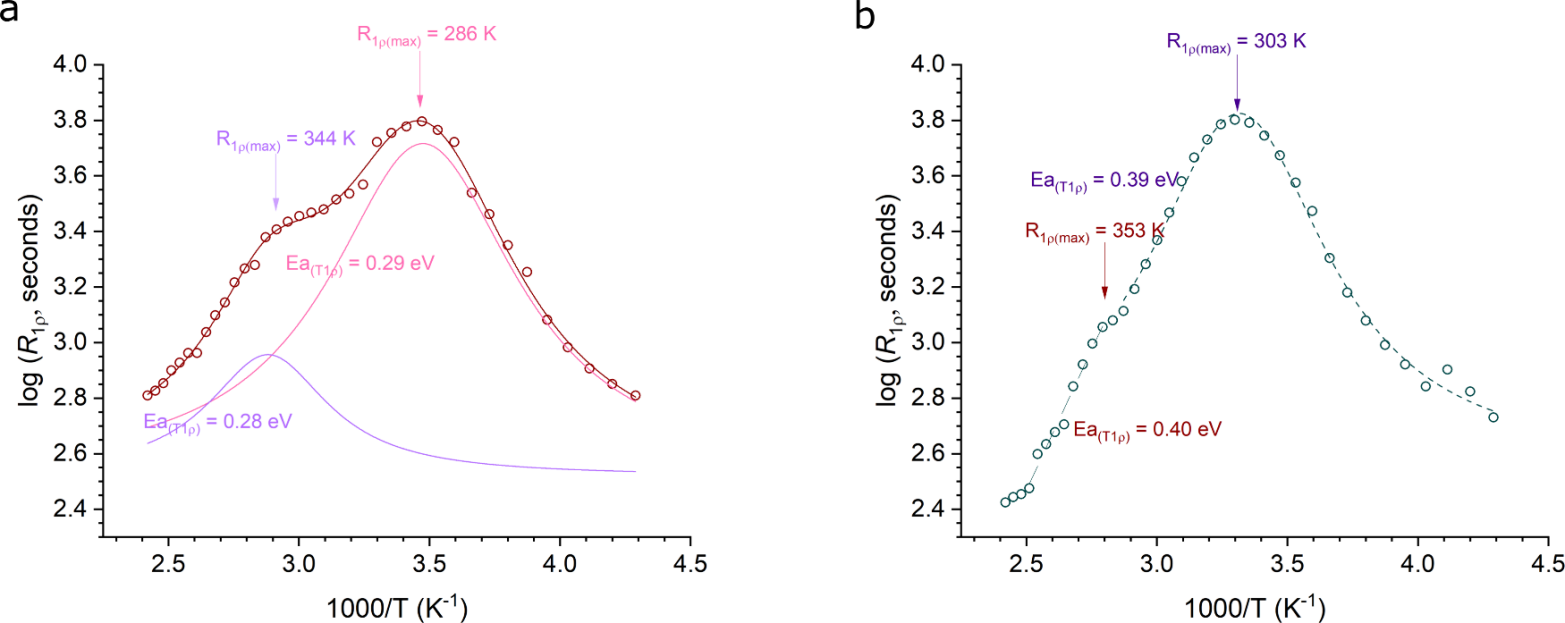
Lithium ion mobility. SLR-NMR R_1ρ_ measurements for a) glass and b) crystalline Li_2_OHBr. Two rate peaks can be seen in each instance. The high-temperature flank activation energies and temperature of the peak maximum are both lower in the glass sample, indicating better lithium dynamics.

In the glass, a high-temperature flank activation energy of ∼0.29 eV is seen for both mobility processes, which is substantially lower than in the crystalline material (∼0.4 eV). The disorder in the glass flattens the energy landscape, making ion mobility more facile. In addition, the two peaks in the glass (R_1ρ(max)_) appear at lower temperatures than in the crystalline material, suggesting that the glass has faster Li-ion dynamics. This is because ion hopping is thermally activated, and a lower temperature peak implies faster hopping at lower thermal energy.

The Meyer-Neldel compensation law is an empirical relationship affecting thermally activated processes, often observed in disordered materials. Specifically, the rule suggests that the activation energy (*E*_a_) and the pre-exponential factor (σ_0_) in an Arrhenius-type expression for conductivity is correlated, such that an increase in one is compensated by an increase in the other, thereby meaning a reduction in activation energy would not necessarily translate to an improvement in conductivity. Our findings indicate both a reduction in activation energy and increased hopping rate in glass samples. These observations suggest that in the case of Li_2_OHBr antiperovskites, enhancements to the Li-ion conductivity are gained from vitrification.

Although the SLR-NMR measurements indicate improved lithium dynamics in the glassy state, longer-range ionic conductivity is unknown, which can be established through electrochemical impedance spectroscopy (EIS). Direct sputtering of blocking electrode contacts onto the glass flakes was found to be challenging due to the small size and fragile nature of the flakes. Instead, pellets of samples can be produced by a conventional cold-pressing approach, in which high pressures are typically required to reduce impacts from particle interfaces. EIS of a pellet of crushed glass, cold-pressed at 370 MPa for 3 minutes, was used to try and obtain measurements of the glass’s ionic conductivity. However, DSC taken on a pressed Li_2_OHBr glass pellet no longer exhibited the glass transition behavior observed in the pristine powder, indicating that crystallization of the samples is occurring under high pressure (Figure S8). This meant that it was not possible to measure an ionic conductivity of the glasses. The unstable nature of these glasses may make them impractical for any potential application as an electrolyte, regardless of their performance. Future work may focus on determining whether bulk samples can be produced without crystallization, potentially by using lower pressing pressures, by optimizing twin-roll quenching conditions to produce larger ribbons, or by exploring alternative synthesis routes such as thin-film techniques. Alternatively, efforts could be directed toward improving the crystallization stability of these glasses, for example, by light doping with traditional glass formers to help “confuse” the structure.

In this paper, glassy Li_2_OHBr, Li_2_OHCl and Li_2_OHCl_0.5_Br_0.5_ are synthesized. Pure glasses appear optically transparent, contain no XRD peaks, and exhibit a glass transition on their first heating cycle. These glasses were found to be challenging to synthesize, requiring extreme cooling rates achievable only by a twin-roll quench process. Synthesis was most successful with the Li_2_OHBr composition. SLR-NMR measurements revealed a lower activation energy and higher hopping frequency in the glassy samples, indicating an improved ionic conductivity over the crystalline state. This improvement in dynamics may be due to the increased free volume in the glassy structure (). It may also be attributable to a reduced ionic interaction of lithium ions with the glass structure, indicated by the more positive peak shift in ^7^Li NMR measurements. In spite of our findings suggesting improvements to lithium dynamics may be gained from the vitrification of Li_2_OHBr, cold-pressing pellets for EIS was found to result in their crystallization. In further work, it would be beneficial to establish processing conditions for bulk glass electrolyte samples.

## Supporting Information Available

Additional experimental details and results including XRD, DSC, SEM, Raman spectroscopy, and NMR (PDF)
