# Supplementary material for: Lithium Antiperovskite-Derived Glass Solid Electrolytes
Source: ACS Mater Lett. 2025 Feb 25;7(4):1187–94. doi: 10.1021/acsmaterialslett.4c02578 (PMC11979957; doi:10.1021/acsmaterialslett.4c02578)
Supplement: Supplementary file 1 — tz4c02578_si_001.pdf [file tz4c02578_si_001.pdf]

Lithium antiperovskite-derived glass solid electrolytes - Supporting Information

Emily Milan[1], Gregory J. Rees[1], Aaron Phillips[2], Cristian Cano[2], Yi Wei[2], Hua Guo[1], Steve Feller[2], and Mauro Pasta*[1]

[1]Department of Materials, University of Oxford, Oxford, OX1 3PH, UK

[2]Department of Physics, Coe College, Cedar Rapids, Iowa, 52402, USA

*Corresponding author: mauro.pasta@materials.ox.ac.uk

Experimental

Synthesis: For synthesis, described in the main text, anhydrous LiOH (98%, Sigma-Aldrich), LiBr ($\geq$ 99%, Sigma-Aldrich) and LiCl ($\geq$ 98%, Alfa Aesar) were used.

Due to the highly hygroscopic nature of the compounds of interest, all work was carried out under inert conditions. Samples were handled in argon-filled gloveboxes (MBraun, H_2O < 0.5ppm, O_2 < 0.5ppm) and were transferred between gloveboxes in sealed glass vials with parafilm around joints, with the exception of the twin-roll quench process and XRD measurements, which were carried out in nitrogen-filled gloveboxes. Utensils and consumables used were dried in a vacuum oven (~1 mbar, 70 ^oC) for a minimum of 2 hours prior to use, and were transferred directly from the oven to the glovebox antechamber.

XRD: XRD measurements were taken on powder samples loaded on a single crystal silicon holder using a Rigaku Miniflex diffractometer (Cu Kα).

DSC: For DSC measurements, a TA Instruments DSC25 was used. A known mass (2 - 5 mg) of sample was hermetically-sealed in aluminium pans under argon atmosphere to avoid air exposure. For measurements, a ramp rate of 5 K/min was used to conduct 3 heating-cooling cycles from -40 ^{o}C to 400 ^{o}C.

SEM, EDX and PFIB: SEM images and EDX were taken on a Thermo Scientific Helios G4 PFIB CXe DualBeam with an Oxford Instruments EDX detector. A Gatan iload vacuum transfer vessel was used to prevent air exposure upon sample loading. Sample cross-sections were prepared using a room-temperature milling and polishing procedure, finishing at a 30 kV, 4 nA ion beam. Little improvement was seen from further polishing steps.

Density Measurements: The density of Li_2OHBr glass flakes was measured using a sink-float method. 3 ml of anhydrous acetone ($\geq$ 99.8 % Sigma-Aldrich, 0.7847 g/ml) and 3 ml of dioodomethane (99 % Sigma-Aldrich, 3.325 g/ml) were initially mixed together in a vial at 25 ^{o}C using a magnetic stirring rod to produce a homogeneous liquid. A glass flake was added which sinks to the bottom. Diiodomethane was added in 0.05 ml increments using a pipette. The vial cap was replaced between additions to avoid volatisation of the acetone, and the sample stirred for $\approx$ 30 seconds. The miscibility of diiodomethane in acetone is high, and so homogeneous solutions are attained easily. The density of the glass flake can be calculated from the ratio of acetone to dioodomethane at the point when the glass flake transitions from sinking to floating. Measurements were taken on 4 glass flakes. No visible reaction occurred to the glass flakes during measurements, and flakes added at the end of the measurement behaved the same as those in solution for the whole measurement, suggesting that no reactions are affecting the measurements.

Raman Spectroscopy: A Renishaw inVia Reflex laser confocal Raman microscope with a 532nm laser and 1800 lines mm^{-1} grating was used to measure powder samples. The instrument operates in air, and so samples were sealed in vials, through which the laser was focused. Scans were initially measured at low powers and observed to check structural changes were not occurring as a consequence of the laser, such as by local heating. Presented spectra were recorded with a laser power of 150 mW and an exposure time of 1 s. Background removal was conducted using the Renishaw WiRE 5.5 software.

NMR: Static solid static ^{7}Li NMR was completed at $\upsilon_0(^7\mathrm{Li})$ = 155.53 MHz (9.45 T) using a 5 mm solution state probe, the temperature was calibrated using $KBr_{(s)}$ and all samples are referenced to $LiCl_{(aq)}$ at 0 ppm.[1, 2] The spin-lock $T_{1\rho}$ relaxometry experiments utilized a 10 kHz B_{1eff}-field that was varied from 1 μs to 100 ms. The data was fitted to $S(t) = I_0[1 - e^{\frac{-t}{T_{1\rho}}}]$. The errors of < 0.5 %, R^2 of 0.99 with normal residual distributions. For MAS ^{7}Li NMR measurements, the magic angle spinning frequency was 10 kHz using a Bruker 4 mm double air bearing probe.

Supporting Information Note 1: Glass Synthesis Attempts

Prior to successful synthesis using the twin-roll quench technique, other approaches were investigated. Initial quenching attempts involved transferring molten sample from the furnace to a thermoelectric cooler ($\sim 5\,^{\circ}\mathrm{C}$). Quenching was also attempted by sealing small masses of antiperovskite in quartz tubes under vacuum, which could then be quenched into a bath of liquid nitrogen outside of the glovebox following melting. This approach has previously been conducted in sulphide glass research [3–6]. Small masses of samples ($\sim$ 100 mg) were used in each instance, to ensure heat transfer through the sample occurred quickly. Nonetheless, highly crystalline antiperovskites were found to form from the resulting XRD patterns. The twin-roll quenching approach achieves significantly higher cooling rates than conventional quenching, through a combination of the thin sheet geometry of the material produced, which provides a high surface-area-to-volume ratio, and the direct contact between the molten material and actively cooled rollers, enabling highly efficient heat extraction.

In addition to quenching attempts, ball milling was investigated as an alternative route to amorphisation, disrupting long-range periodicity through a combination of mechanical strain and localised heat induced by the high-impact collisions. This approach became popular in sulphide glass research to enable synthesis of compositions with poor glass-forming ability [7–11]. It was hoped that ball milling might be useful in the antiperovskite compositions, which are similarly challenging to vitrify by alternative methods. Milling was carried out in a planetary ball mill (Fritsch Pulverisette 7 Premium) under argon atmosphere using 1 g of Li_2OHBr and zirconia balls in a ball-to-powder ratio of 13:1. Cycles of 3 minutes milling at 500 rpm followed by 7 minutes rest were used to allow dissipation of heat built up during the milling steps. XRD measurements were taken on sample after 30, 55, 70 and 120 hours effective milling. Although a reduction in peak intensity and crystallite size was observed, samples did not become amorphous during this extended period of milling.

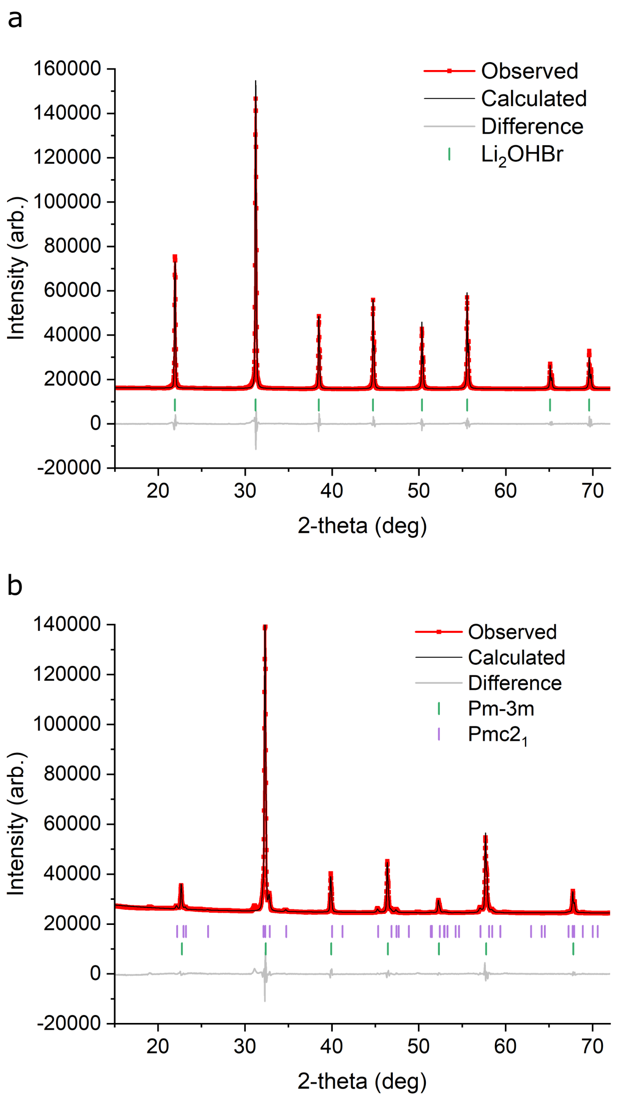

Figure S1: Pawley refinements of crystalline diffraction patterns shown in the main text. a) Li_2OHBr. Cubic (Pm-3m) a = 4.049 Å. R_{wp} = 9.73 %. b) Li_2OHCl. Cubic (Pm-3m) a = 3.906 Å. Orthorhombic ($Pmc2_1$) a = 3.874 Å, b = 3.829 Å, c = 7.997 Å. R_{wp} = 7.96 %. Bragg peak positions belonging to each crystal structure are indicated with ticks [12]

Sample	Space Group	Lattice Parameter (Å)			R_{wp} (%)
		a	b	c	
Li_2OHBr	Pm-3m	4.049	4.049	4.049	9.73
Li_2OHCl	Pm-3m	3.906	3.906	3.906	7.96
Li_2OHCl	$Pmc2_1$	3.874	3.829	7.997	7.96

Table S1: Unit cell parameters of crystalline Li_2OHBr and Li_2OHCl, determined from the Pawley refinements shown in Figure S1.

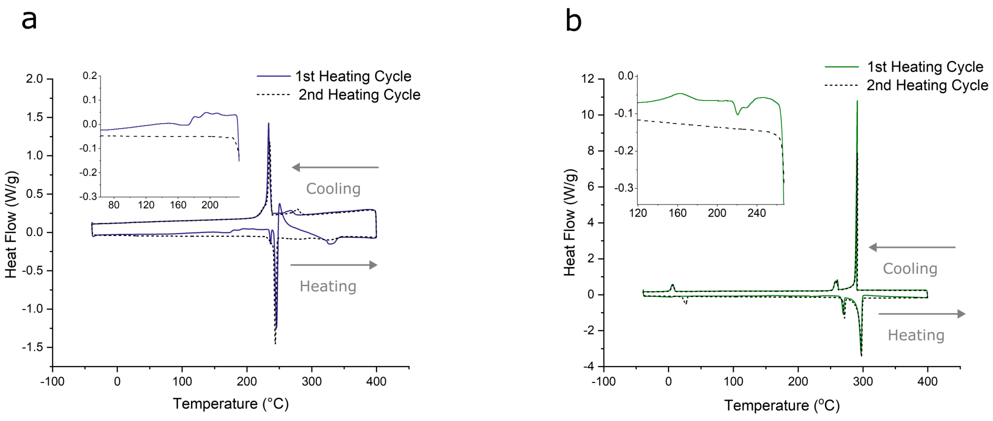

Figure S2: Loss of the Glassy State. Two DSC heating-cooling cycles of glassy a) Li_2OHBr and b) Li_2OHCl, showing the disappearance of the glass transition after the initial heating. A negative heat flow corresponds to an endothermic response.

Supporting Information Note 2: Mixed Phase 'Glass-Ceramics'

Although it was possible to obtain pure Li_2OHBr and Li_2OHCl glass samples, it was typical for there to be some crystalline material in the samples, even after optimisation of the synthesis conditions. These samples did not appear fully transparent, and XRD patterns of samples showed Bragg peaks corresponding to the antiperovskite phase with reduced intensity compared to measurements of homologue crystalline samples taken under the same conditions (Figure S3a, d-f). Transmission electron microscopy (TEM) electron diffraction (ED) of a crushed partially-transparent Li_2OHBr flake showed a mixture of amorphous and crystalline regions (Figure S3b,c). For the $Li_2OHCl_{0.5}Br_{0.5}$ samples, it was only possible to produce these mixed phase 'glass-ceramics' rather than pure glasses. Using an internal standard method, the amorphous fraction in these samples was estimated to be around 70 % (see Supporting Information Note 3). Interestingly, the glass transition appears at $\approx 130^oC$, around ~ 0.5 T_m (Figure S3g). This is lower than for the other samples, which were close to the typical 2/3 T_m seen in inorganic glasses [13]. This may explain why it was more difficult to produce pure glasses of the $Li_2OHCl_{0.5}Br_{0.5}$ composition [14].

Synthesis was most successful using the Li_2OHBr composition. Nevertheless, even after optimisation of the synthesis conditions, the amorphous content of different yields was found to vary vastly. A relationship is found in metallic glasses, whereby glass-forming ability improves with a higher 'reduced glass transition temperature', equivalent to T_g/T_l where T_l is the liquidus temperature [15]. Since T_g typically varies slowly with composition, this can be achieved with eutectic, or near-eutectic, stoichiometries. The eutectic composition on the LiBr-LiOH phase diagram (40 mol % LiOH) was investigated to see whether improved glass forming could be achieved, however an improvement was not observed. Consequently, in subsequent characterisation the best quality Li_2OHBr glass flakes were selected based on appearance, to try and obtain measurements for pure glasses. It is likely that a small amount of crystalline phase may be present.

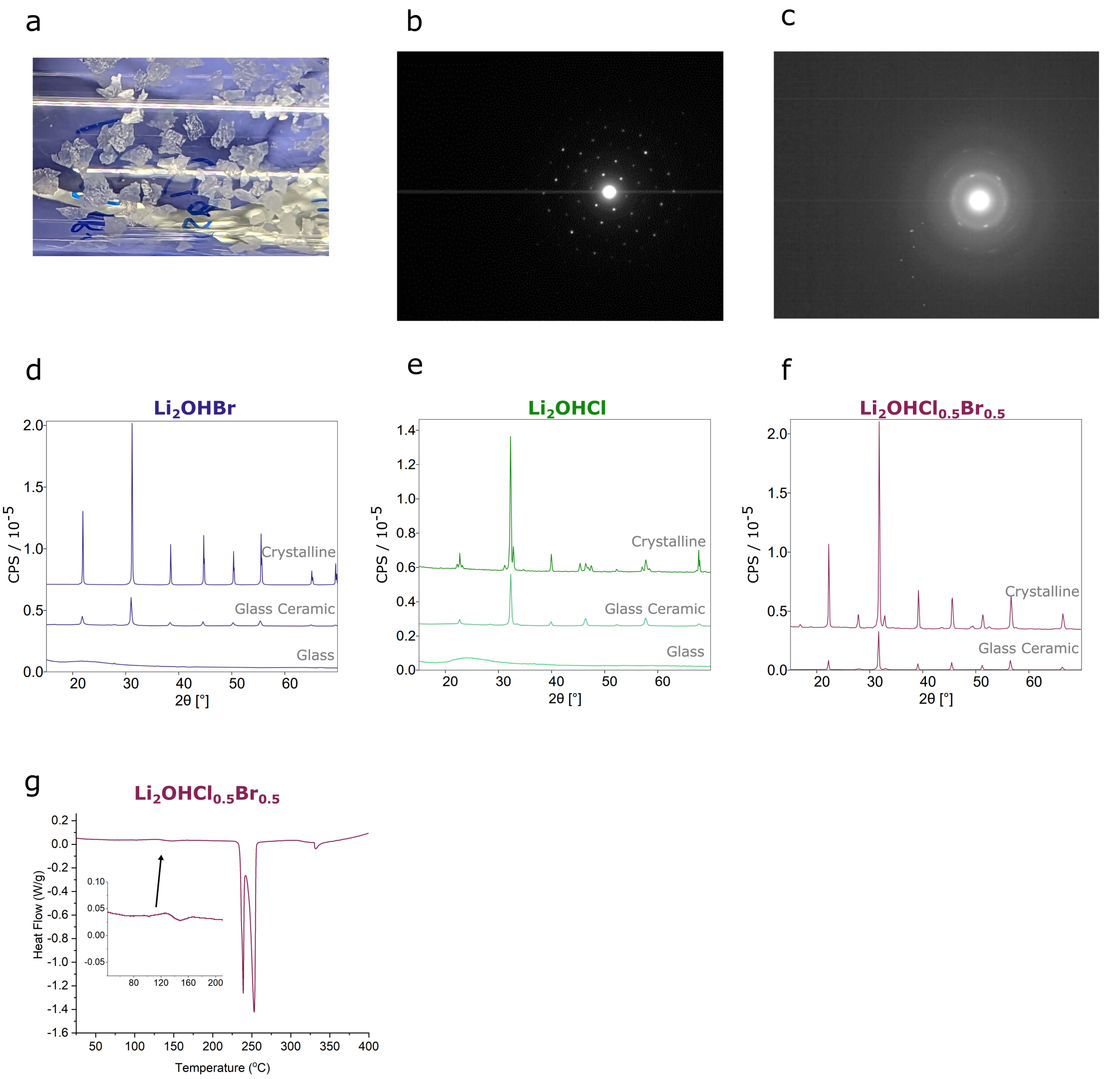

Figure S3: Glass-Ceramics. a) Photograph of partially-transparent glass-ceramic samples, stored in a vial. b) TEM ED pattern of a crystalline region of an Li_2OHBr flake showing sharp discreet spots. c) TEM ED pattern of a glassy region of an Li_2OHBr flake showing diffuse rings, indicative of an amorphous structure. d-f) XRD patterns showing varying crystallinity in Li_2OHBr, Li_2OHCl and $Li_2OHCl_{0.5}Br_{0.5}$ flakes respectively. g) DSC measurement of glass-ceramic $Li_2OHCl_{0.5}Br_{0.5}$ showing evidence of a glass transition.

Supporting Information Note 3: Glass Fraction Estimation

To estimate the amorphous fraction in samples, an internal standard method was used [16]. A known mass of an internal standard was mixed into the sample and the XRD pattern measured. The peaks were assigned to each of the phases, followed by background subtraction and peak integration carried out using PDXL software. From this, the amorphous fraction in the sample can be estimated by considering the observed relative intensities of the phases (i.e. the crystalline response) to the known ratio of the two samples. Equations 1 and 2 can be used to this outcome:

$$\left(\frac{I_{sample}}{I_{standard}}\right) \times \text{wt fraction standard} = F_{cryst} \tag{1}$$

$$F_{amorph} = 1 - F_{cryst} \tag{2}$$

Both a silicon nanopowder and LiCl granule were explored as a standard for this process. These materials have few XRD peaks, distinct from the antiperovskite peaks, and did not react with the samples upon mixing. The low masses of glasses available for testing meant that homogeneous mixtures could not be easily produced when using the LiCl granules, even using a mortar and pestle for the mixing process. The fine nature of the nanopowder meant that this was not a problem with the silicon standard. However, the nanopowder contains a large fraction of amorphous material, which needed to be accounted for. Consequently, the crystalline fraction in the silicon nanopowder was estimated through an internal standard method with LiCl. The availability of these materials meant that larger batches with better mixing could be done. As shown in Table S2, this gave an amorphous fraction of 68 % ± 5 % in the silicon nanopowder, which could then be accounted for in the glass internal standard calculations by multiplying $I_{standard}$ by $\frac{100}{100-68}$. This gave amorphous contents between 64 % and 71 % for the $Li_2OHCl_{0.5}Br_{0.5}$ glass ceramics (Table S3).

Table S2: Intensities determined from XRD of Si-LiCl mixtures, used to establish the amorphous content in silicon nanopowder. The standard refers to LiCl and the sample to silicon.

Sample	Wt % Standard	Sample Intensity / CPS	Standard Intensity / CPS	Sample Amorphous Content %
1	35.24	21382	30223	64.8
2	55.46	17663	34111	71.3
**Average**				**68.0 ± 4.6**

Table S3: Intensities determined from XRD of $Li_2OHCl_{0.5}Br_{0.5}$ - Si mixtures, along with the 'corrected' standard intensity accounting for the 68 % amorphous content in the silicon nanopowder. Estimations of the amorphous content in glass-ceramic samples are calculated using the approach outlined in Supporting Information Note 3.

**Sample**	**Wt % Standard**	**Intensity**			**Sample Amorphous Content %**
		**Sample / CPS**	**Standard / CPS**	**Corrected Standard / CPS**	
1	9.05	63124	6459	20184	71.7
2	18.57	74275	12352	38624	64.3
3	11.10	50061	5372	16799	66.9

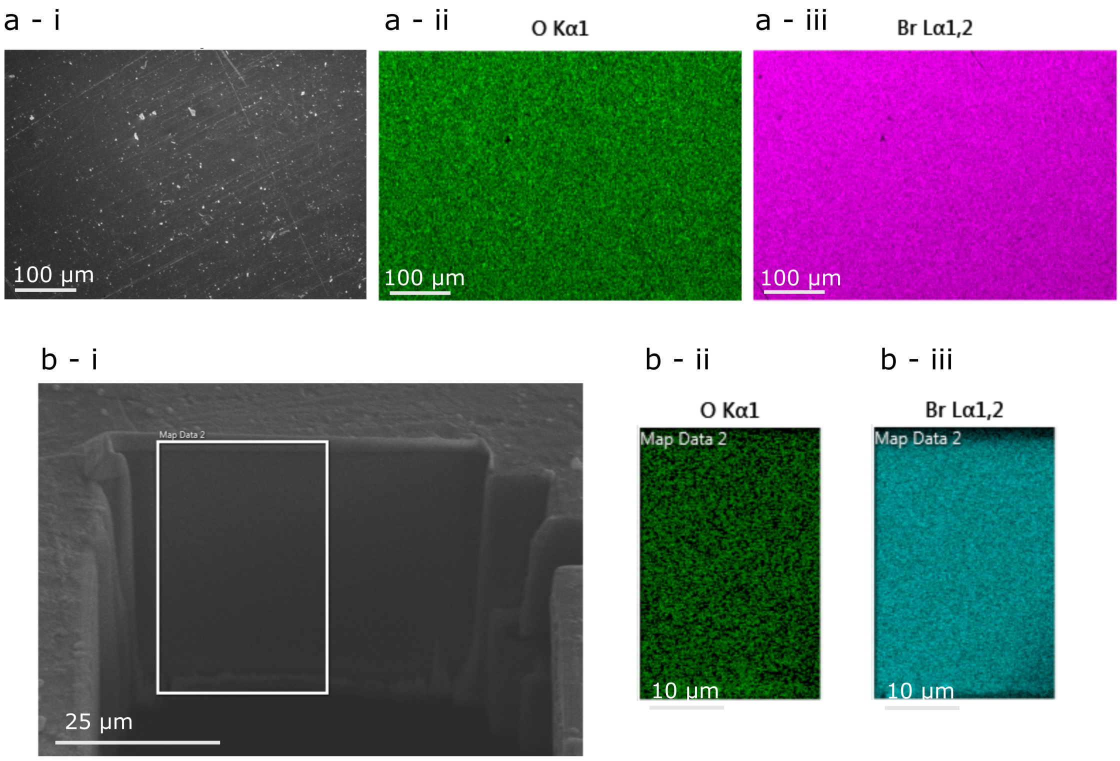

Figure S4: SEM images and corresponding EDX mapping of the a) surface, and b) PFIB cross-section, of a glass Li_2OHBr flake. Note that white specs in a-i correspond to surface contamination.

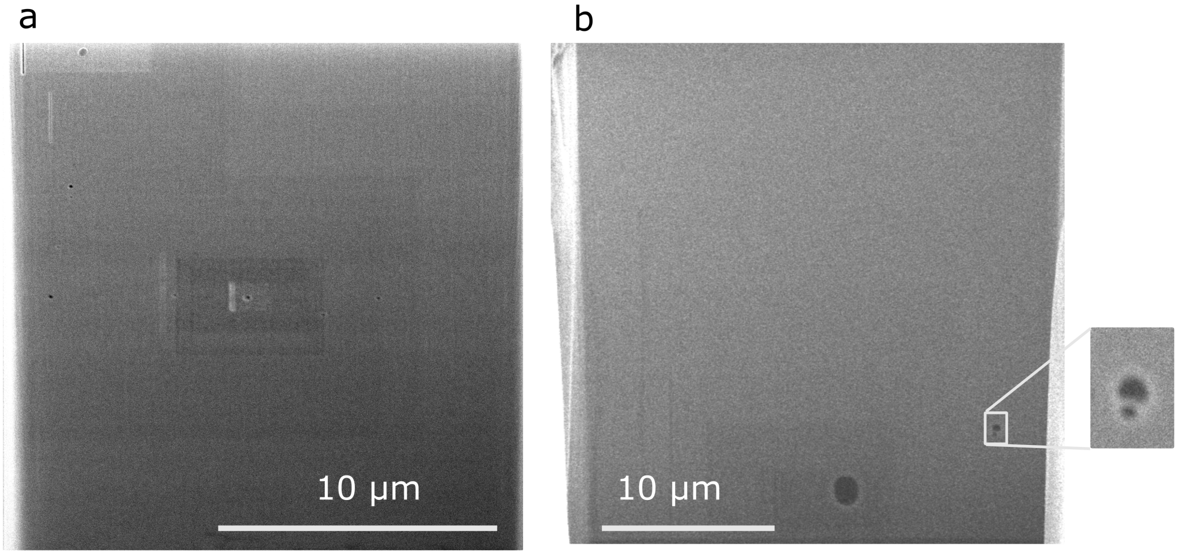

Figure S5: Cross-sectional PFIB images of Li_2OHBr glass flakes, showing low porosity in each case. a) the same region as shown in Figure 2b. Here clear halos can be seen around the pores, supporting that the features are pores, opposed to inclusions of another phase. Note that the boxes present correspond to regions of beam damage arising from imaging. b) A different flake, showing a few larger pores, but still a high relative density.

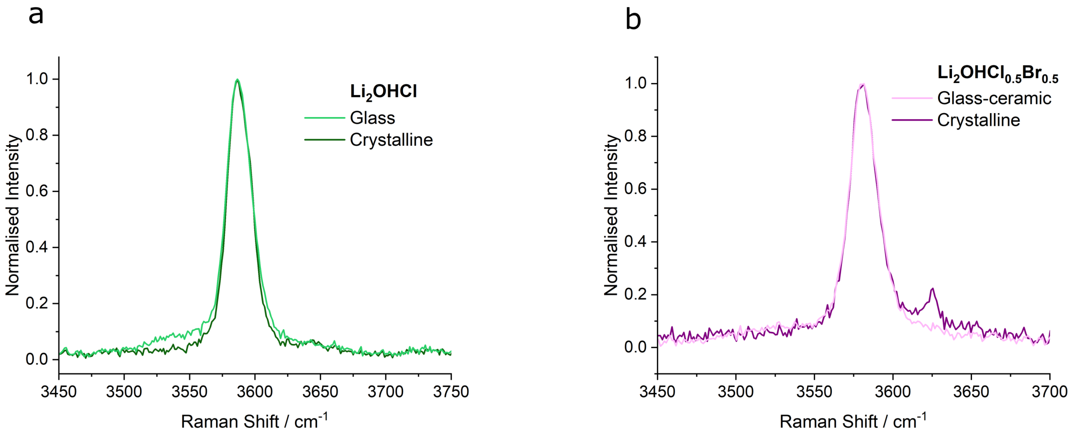

Figure S6: Raman Spectroscopy measurements taken between 3450 and 3750 cm^{-1} on glassy and crystalline samples. a) Li_2OHCl showing a single peak at 3579 cm $^{-1}$, and b) $Li_2OHCl_{0.5}Br_{0.5}$ showing a main peak at 3582 cm^{-1} with a small additional peak in the crystalline sample at 3626 cm $^{-1}$. The O-H bonding environment is largely the same in the glassy and crystalline state for each composition.

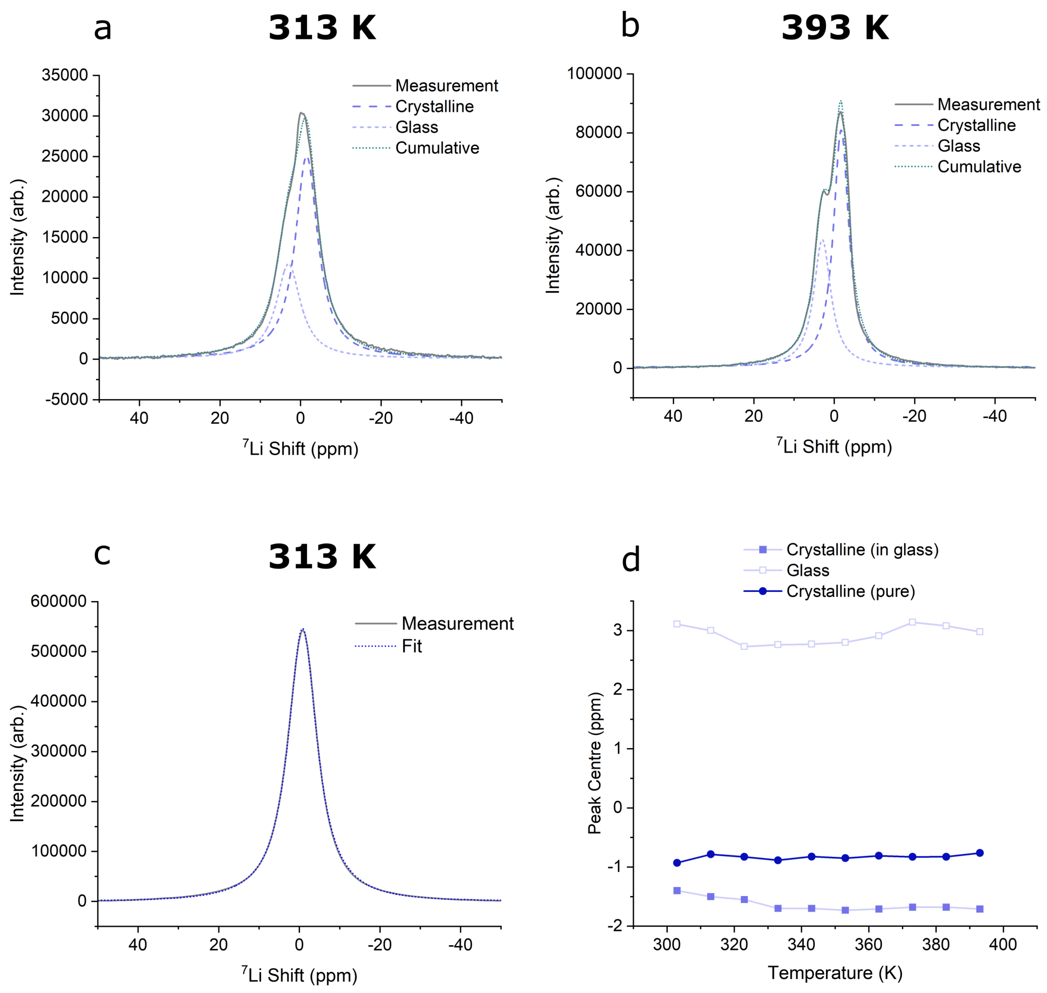

Figure S7: Peak model applied to the ^{7}Li MAS NMR spectra. a) Glass-ceramic at 313 K, b) Glass-ceramic at 393 K and c) Crystalline sample at 313 K. d) Peak centres plotted as a function of temperature. Curve fitting was carried out using 2 Gaussian-Lorentzian peaks. A small shift in position of the crystalline peak is seen between the glass-ceramic and pure crystalline sample due to the differing environment.

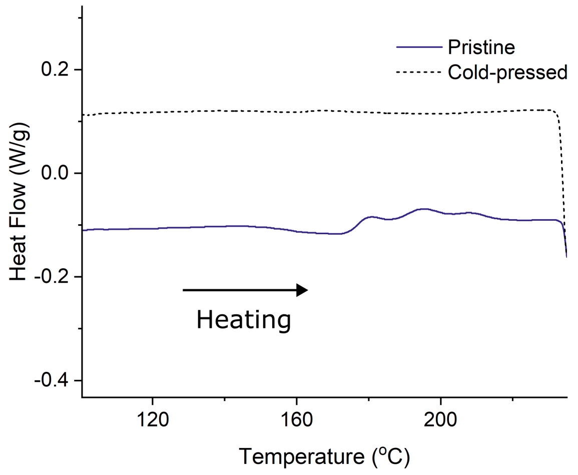

Figure S8: Magnified curve showing that the glass transition has been lost in glassy Li_2OHBr upon cold-pressing. A negative heat flow corresponds to an endothermic response.

References

[1] Kent R. Thurber and Robert Tycko. Measurement of sample temperatures under magic-angle spinning from the chemical shift and spin-lattice relaxation rate of 79Br in KBr powder. *Journal of Magnetic Resonance*, 196(1):84–87, 1 2009. ISSN 10907807. doi: 10.1016/j.jmr.2008.09.019.

[2] Robin K Harris, Edwin D Becker, Sonia M Cabral De Menezes, Robin Goodfellow, and Pierre Granger. NMR Nomenclature: Nuclear Spin Properties and Conventions for Chemical Shifts (IUPAC Recommendations 2001). Technical report, 2001. URL `http://www.iupac.org/publications/pac/2001/7311/7311x1795.html`.

[3] Rene Mercier, Jean-Pierre Malugani, Bernard Fahys, and Guy Robert. Superionic Conduction in Li2S-P2S5-LiI Glasses. *Solid State Ionics*, 5:663–666, 1981.

[4] M Menetrier, A Levasseur, C Delmas, Laboratoire De Chimie, and Universit De Bordeaux L. New Secondary Batteries for Room Temperature Applications Using a Vitreous Electrolyte. *Solid State Ionics*, 14:257–261, 1984.

[5] John H Kennedy and Yuan Yang. Glass-Forming Region and Structure in SiS2-Li2S-LX (X = Br, I). *Journal of Solid State Chemistry*, 257:252–257, 1987.

[6] S Kondo, K Takada, and Y Yamamura. New Lithium Ion Conductors Based on Li2S-SiS2 System. *Solid State Ionics*, 56:1183–1186, 1992.

[7] Masahiro Tatsumisago, Hideki Yamashita, Akitoshi Hayashi, Hideyuki Morimoto, and Tsutomu Minami. Preparation and structure of amorphous solid electrolytes based on lithium sulfide. *Journal of Non-Crystalline Solids*, 274:30–38, 2000.

[8] A Hayashi, S Hama, H Morimoto, M Tatsumisago, and T Minami. Preparation of Li 2 S–P 2 S 5 Amorphous Solid Electrolytes by Mechanical Milling. *Journal of the American Ceramic Society*, 84 (2):477–479, 2001.

[9] Satoshi Ujiie, Akitoshi Hayashi, and Masahiro Tatsumisago. Preparation and ionic conductivity of (100x)(0.8Li2S·0.2P2S5)·xLiI glass–ceramic electrolytes. *Journal of Solid State Electrochemistry*, pages 675–680, 2013. doi: 10.1007/s10008-012-1900-7.

[10] Akihiro Yamauchi, Atsushi Sakuda, Akitoshi Hayashi, and Masahiro Tatsumisago. Preparation and ionic conductivities of (100-x)(0.75Li2S-0.25P2S5)xLiBH4 glass electrolytes. *Journal of Power Sources*, 244:707–710, 2013. doi: 10.1016/j.jpowsour.2012.12.001.

[11] Yoshikatsu Seino, Tsuyoshi Ota, Kazunori Takada, Akitoshi Hayashi, and Masahiro Tatsumisago. A sulphide lithium super ion conductor is superior to liquid ion conductors for use in rechargeable batteries. *Energy and Environmental Science*, 7(2):627–631, 2014. ISSN 17545706. doi: 10.1039/c3ee41655k.

[12] Anucha Koedtruad, Midori Amano Patino, Noriya Ichikawa, Daisuke Kan, and Yuichi Shimakawa. Crystal structures and ionic conductivity in Li2OHX (X = Cl, Br) antiperovskites. *Journal of Solid State Chemistry*, 286:121263–121267, 6 2020. ISSN 1095726X. doi: 10.1016/j.jssc.2020.121263.

[13] S Sakka and J D Mackenzie. Relation Between Apparent Glass Transition Temperature and Liquidus Temperature for Inorganic Glasses. *Journal of Non-Crystalline Solids*, 6:145–162, 1971.

[14] David Turnbull. Under What Conditions Can A Glass Be Formed? *Contemporary Physics*, 10(5): 473–488, 9 1969. ISSN 13665812. doi: 10.1080/00107516908204405.

[15] Z P Lu, H Tan, Y Li, and S C Ng. The Correlation Between Reduced Glass Transition Temperature and Glass Forming Ability of Bulk Metallic Glasses. *Scripta Materialia*, 42(7):667–673, 2000.

[16] Katsumasa Yasukawa, Yoshitake Terashi, and Akira Nakayama. Crystallinity Analysis of Glass-Ceramics by the Rietveld Method. *Journal of the American Ceramic Society*, 81(11):2978–2982, 1998.
